# Clinical value and role of microRNA-29c-3p in sepsis-induced inflammation and cardiac dysfunction

**DOI:** 10.1186/s40001-021-00566-y

**Published:** 2021-08-10

**Authors:** Bingyu Zhang, Lin Yu, Ying Sheng

**Affiliations:** 1 Department of Critical Care Medicine, Gongli Hospital of Pudong New Area of Shanghai, Shanghai, 200135 China; 2 Department of Emergency and Critical Care Medicine, Shanghai Pudong Hospital, Fudan University Pudong Medical Center, No. 2800, Gongwei Road, Pudong district, Shanghai, 201399 China

**Keywords:** Sepsis, miR-29c-3p, Diagnosis, Cardiac dysfunction, Inflammation

## Abstract

**Background:**

The goal of this study was to investigate the diagnostic value of miR-29c-3p in sepsis and its role in sepsis-induced inflammatory response and cardiac dysfunction.

**Methods:**

Serum level of miR-29c-3p was detected by qRT-PCR. The ROC curve was used to evaluate the diagnostic value of miR-29c-3p for Sepsis. The cecal ligation and puncture method (CLP) was used to establish a rat sepsis model. To assess cardiac function, left ventricular systolic pressure (LVSP), left ventricular end diastolic pressure (LVEDP) and maximum rate of rise/fall of left ventricle pressure (± d*p*/d*t*_max_) in different experimental groups were detected, and the serum cardiac troponin I (cTnI), creative kinase isoenzyme MB (CK-MB) were measured by ELISA. Meanwhile, TNF-α, IL-1β, and IL-6 were detected by ELISA to assess the level of inflammatory response in animals.

**Results:**

miR-29c-3p level was upregulated in sepsis patients. ROC curve revealed that miR-29c-3p had the ability to distinguish sepsis patients from healthy controls. Cardiac dysfunction and inflammation were observed in sepsis rat, which were characterized by the decrease of LVSP and + d*p*/d*t*_max_, the increase of LVEDP, − d*p*/d*t*_max_, cTnI, CK-MB, TNF-α, IL-1β, IL-6. All effects were reversed by the injection of miR-29c-3p antagomir. Logistics regression analysis manifested miR-29c-3p is an independent factor in the occurrence of cardiac dysfunction in sepsis patients.

**Conclusions:**

miR-29c-3p has potential as a biomarker for the diagnosis of sepsis, and inhibition of miR-29c-3p expression in animal models reduced sepsis-induced cardiac dysfunction and inflammatory response.

## Background

In recent years, modern medical diagnosis and treatment technology has advanced by leaps and bounds, but sepsis is still an important death cause for patients in the intensive care unit (ICU). It is estimated that about 5.3 million people die from sepsis every year in the world [1]. The relevant studies on sepsis are still a research hotspot in the medical field. Sepsis 3.0 defines sepsis as a life-threatening condition resulting from a dysregulation of the body’s response to infection [2]. The essence of sepsis is the excessive inflammatory reaction of the body caused by infection [3]. However, due to its complex pathophysiological mechanism, it involves multiple links such as inflammation and immunity [4], and it often takes several days to complete bacteriological evidence [1, 5]. Therefore, the diagnosis and treatment of sepsis is still difficult due to the lack of specificity of rapid diagnosis and treatment.

MiRNAs are a class of non-coding RNAs (ncRNAs) with gene regulation function and approximately 19–24 nucleotides in length [6, 7]. By recognizing and combing with the 3′-non-transcriptional regions (3′-UTR) of target mRNA, miRNAs guide the silencing complex to degrade mRNA or block its translation, thus affecting the expression of proteins in the next step [8]. Many miRNAs have been found to have specific diagnostic value in certain diseases. For example, in the myocardial cells of the sepsis mouse model, miR-223 expression was significantly down-regulated, and the further deletion of miR-223 aggravated the cardiac insufficiency and inflammatory response of the sepsis mice [9]. miR-29c-3p belongs to the miR-29 family and was elevated in fibroblasts and VSMCs [10]. A recent study showed abnormally high miR-29c-3p in inflammatory diseases, such as adult-onset Still’ s disease (AOSD) patients [11]. Considering that there are relatively few studies on the relationship between miR-29c-3p and sepsis, the specific mechanism remains to be explored.

In this present study, serum miR-29c-3p in sepsis patients has been detected and the clinical value of miR-29c-3p as a diagnostic marker of sepsis has also been assessed. At the same time, we further evaluated the effects of sepsis on cardiac function and inflammatory response in rats by establishing sepsis rat model.

## Materials and methods

### Participants and samples

The study has been authorized by the Ethics Committee of Gongli Hospital of Pudong New Area of Shanghai, and each participant and his/her family members have been informed and signed a written informed consent. 86 sepsis patients admitted to the ICU of this hospital were recruited and the diagnostic criteria for sepsis follow the international diagnostic criteria for Sepsis 3.0 issued by SCCM/ESICM in 2016 [12]. Excluded participants met the following criteria: patients with chronic diseases, hematologic disease, and malignant tumors; people with congenital immune deficiency; pregnant or lactating women. Besides, 85 healthy people undergoing health examination in the same period were selected as the healthy controls, who had no major diseases or other abnormalities. Venous bloods were gathered from patients within 24 h of admission to the ICU and the APACHE-II score and SOFA score were estimated. The general clinical and clinicopathological indicators of the participants were collected and recorded.

### Construction of sepsis rat model

Experiments involving animals were allowed by the Medical Ethics Committee of Gongli Hospital of Pudong New Area of Shanghai and were followed with the guidelines for the care and use of laboratory animals. 40 adult male SD rats weighing 250 g to 300 g were collected from Shanghai Animal Center. Before the experiment, they lived under suitable conditions (23 ± 1 °C, light/dark cycle) and were allowed to eat and drink freely. As mentioned earlier, cecal ligation and puncture (CLP) was used to replicate the animal model of sepsis [13]. First, pentobarbital sodium (50 mg/kg) was used to anesthetize rats. A 2 cm surgical incision was made in the medioventral line of the rats, and the cecum was located after incision of the skin. Next, 1/3 of the cecum was ligated with line 3, and 3 punctures were performed with the needle of the No. 18 syringe at the center of the ligation end, while avoiding damage to blood vessels. Finally, the cecum was placed in the abdominal cavity and the skin was sutured with sutures 4. Subcutaneously injected normal saline (50 mL/kg) immediately after the operation for antishock. The control group received the same surgical treatment without CLP.

### Grouping and treatment

SD rats were divided into control group (without CLP-treatment, injected with normal saline), CLP group (with CLP-treatment, injected with normal saline), miR-29c-3p NC group (with CLP-treatment, 10 μg miR-29c-3p NC was injected intravenously 24 h before surgery) and miR-29c-3p antagomir group (with CLP-treatment, 10 μg miR-29c-3p antagomir was injected intravenously 24 h before surgery) with 10 rats in each group. The miR-29c-3p NC and antagomir used in this study were synthesized and provided by GenePharma (Shanghai, China).

### Measurement of hemodynamic indexes

The hemodynamic parameters of rats in each group including left ventricular systolic pressure (LVSP), left ventricular and end-diastolic pressure (LVEDP) and maximum rate of change in left ventricular pressure (± d*p*/d*t*_max_) were monitored by MFLab 3.01 software on FDP-1 HRV and BRS system to evaluate the cardiac function of model rats.

### Detection of cardiac function and inflammatory indexes

The venous blood of rats was collected for the detection of biochemical indexes. Serum level of cardiac troponin I (cTnI) and creative kinase isoenzyme MB (CK-MB), as well as tumor necrosis factor α (TNF-α), interleukin 6 (IL-6), interleukin 1β (IL-1β) were measured by Enzyme-linked immunosorbent assay (ELISA) to evaluate the cardiac function and inflammatory response of rats in each group.

### Detection of miR-29c-3p expression levels

qRT‑PCR method detected the level of miR-29c-3p. RNAs were extracted from serum using TRIzol, and the RNAs were reverse transcribed into cDNA by a Prime Script™ RT reagent Kit based on the product specification. Subsequently, PCR analysis was carried out using miScript SYBR® Green PCR kit on the PCR System. U6 was defined as internal reference, and the level of miR-29c-3p was calculated by normalization of U6 in 2^−ΔΔCt^ methods.

### Statistical analysis

SPSS and GraphPad Prism were used for statistical analysis. The student *t*-test or one-way ANOVA was used for comparison between groups, and the Chi-square test was used for comparison of classified variables. The correlation between clinical indicators and miR-29c-3p level was analyzed by Pearson correlation coefficient. Logistic regression analysis was used to estimate the influence of different factors on cardiac dysfunction in sepsis patients. *P* < 0.05 represented a significant difference.

## Results

### Clinical characteristics of sepsis patients

The study involved 171 participants, and the results of their clinical information comparison were shown in Table 1. There were no significant differences between the two groups in age, gender, and body mass index (BMI) (*P* > 0.05). The Scr, Albumin, WBC, CRP and PCT levels in sepsis patients were significantly different from those in healthy controls (*P* < 0.001). Besides, the APACHE II score and SOFA score of the sepsis patient were (11.10 ± 4.00) and (5.05 ± 1.60).

**Table 1:** Clinical characteristics of the subjects

Parameter	Subjects (*N* = 171)		*P* value
	Health individuals (*n* = 85)	Sepsis patients (*n* = 86)	
Age (year)	57.18 ± 10.82	54.56 ± 12.91	0.153
Gender (male/female)	40/45	38/48	0.706
BMI (kg/m^2^)	20.95 ± 2.92	21.83 ± 2.97	0.053
Scr (mg/dL)	1.03 ± 0.28	1.60 ± 0.41	0.000
Albumin (g/L)	42.51 ± 5.84	27.79 ± 4.77	0.000
WBC (× 10^9^/L)	7.21 ± 1.51	15.78 ± 6.38	0.000
CRP (mg/L)	5.51 ± 2.80	79.53 ± 17.20	0.000
PCT (ng/mL)	0.05 ± 0.02	11.10 ± 4.00	0.000
APACHE II score	–	12.19 ± 3.45	–
SOFA score	–	5.05 ± 1.60	–

Data are expressed as n or mean ± standard deviation

*BMI* body mass index, *Scr* serum creatinine, *WBC* white blood cell, *CRP* C-reactive protein, *PCT* procalcitonin, *APACHE* acute physiology and chronic health evaluation, *SOFA* sequential organ failure assessment

### Serum level of miR-29c-3p in sepsis patients

**Fig. 1:**
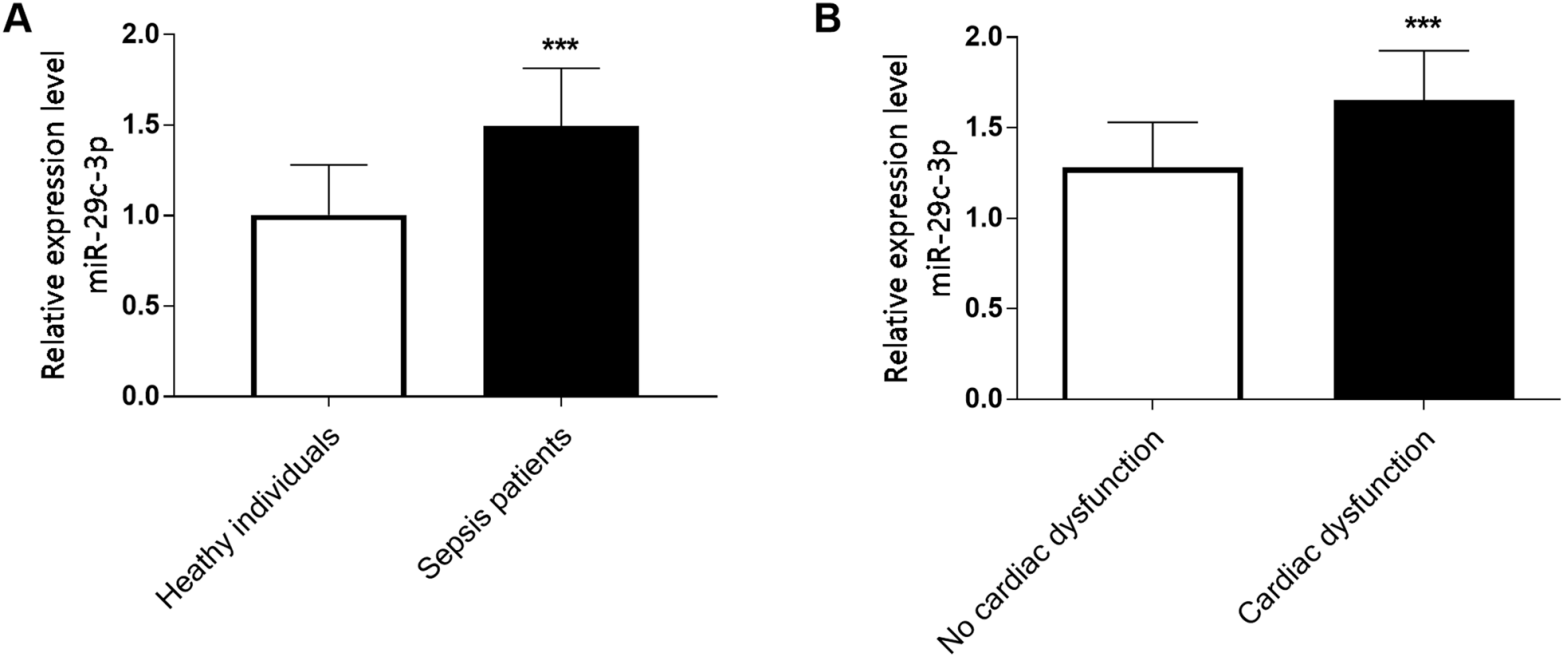
Serum miR-29c-3p level in different populations was analyzed by qRT-PCR. **A** miR-29c-3p level in sepsis patients was increased. **B** miR-29c-3p level in sepsis with cardiac dysfunction was significantly upregulated compared with sepsis without cardiac dysfunction. (***P < 0.001)

qTR-PCR results showed that serum miR-29c-3p level in sepsis patients was significantly upregulated in comparison to healthy controls (Fig. 1A, *P* < 0.001). Furthermore, serum miR-29c-3p level in sepsis patients with cardiac dysfunction was augmented remarkably than that in sepsis patients without cardiac dysfunction (Fig. 1B, *P* < 0.001), suggesting that abnormal miR-29c-3p level was involved in the occurrence of sepsis, and high level of miR-29c-3p expression was associated with the condition of sepsis.

### Correlation analysis of miR-29c-3p and clinicopathological indicators of sepsis

Pearson correlation coefficient analysis revealed that the levels of CRP and PCT, as well as APACHE II score and SOFA score were positively correlated with miR-29c-3p in sepsis patients (Table 2, *P* < 0.001), indicating that the level of miR-29c-3p is positively correlated with the severity of sepsis.

**Table 2:** The relation of miR-29c-3p expression with the clinical variables

Parameters	Correlation (*r*)	*P* value
Age (year)	0.045	0.682
Gender (male/female)	0.152	0.163
BMI (kg/m^2^)	0.131	0.228
Scr (mg/dL)	0.134	0.218
Albumin (g/L)	0.063	0.564
WBC (× 10^9^/L)	0.137	0.207
CRP (mg/L)	0.387	0.000
PCT (ng/mL)	0.474	0.000
APACHE II score	0.459	0.000
SOFA score	0.427	0.000

*BMI* body mass index, *Scr* serum creatinine, *WBC* white blood cell, *CRP* C-reactive protein, *PCT* procalcitonin, *APACHE* acute physiology and chronic health evaluation, *SOFA* sequential organ failure assessment

### Analysis of the diagnostic value of miR-29c-3p in sepsis

**Fig. 2:**
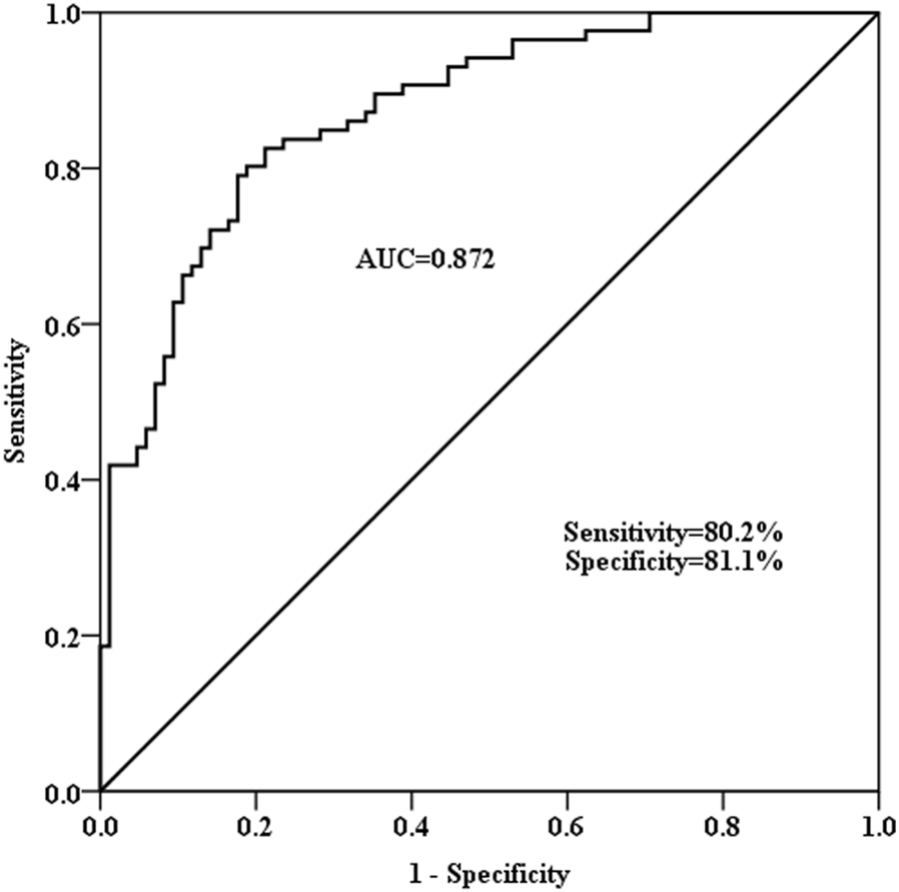
ROC curve analysis

ROC curve was established to evaluate the diagnostic significance of miR-29c-3p in sepsis. In Fig. 2, it revealed that the AUC value was 0.872 with a sensitivity of 80.2% and specificity of 81.1%, suggesting that serum miR-29c-3p is of high diagnostic value in sepsis.

### Association of miR-29c-3p levels with the occurrence of cardiac dysfunction

Based on the cardiac function, patients were divided into normal cardiac function group (*n* = 37) and cardiac dysfunction group (*n* = 49). Subsequently, Logistics regression analysis evaluated the relationship between miR-29c-3p levels and the occurrence of cardiac dysfunction. It suggested that miR-29c-3p was an independent factor in the occurrence of cardiac dysfunction in sepsis patients (Table 3, OR = 3.905, 95% CI = 1.410–10.817, *P* = 0.009).

**Table 3:** Association of different variables with the occurrence of cardiac dysfunction

Parameter	OR	95% CI	*P* value
Age (year)	1.804	0.660–4.930	0.250
Gender (male/female)	1.161	0.445–3.029	0.761
BMI (kg/m^2^)	1.200	0.455–3.166	0.713
Scr (mg/dL)	1.051	0.381–2.897	0.923
Albumin (g/L)	0.505	0.179–1.429	0.198
WBC (× 10^9^/L)	1.590	0.607–4.166	0.346
CRP (mg/L)	1.194	0.428–3.333	0.735
PCT (ng/mL)	1.002	0.373–2.695	0.996
APACHE II score	1.146	0.429–3.057	0.786
SOFA score	1.775	0.581–5.420	0.314
MiR-29c-3p	3.905	1.410–10.817	0.009

*BMI* body mass index, *Scr* serum creatinine, *WBC* white blood cell, *CRP* C-reactive protein, *PCT* procalcitonin, *APACHE*, acute physiology and chronic health evaluation, *SOFA* sequential organ failure assessment

### Effects of miR-29c-3p on cardiac function in animal model

**Fig. 3:**
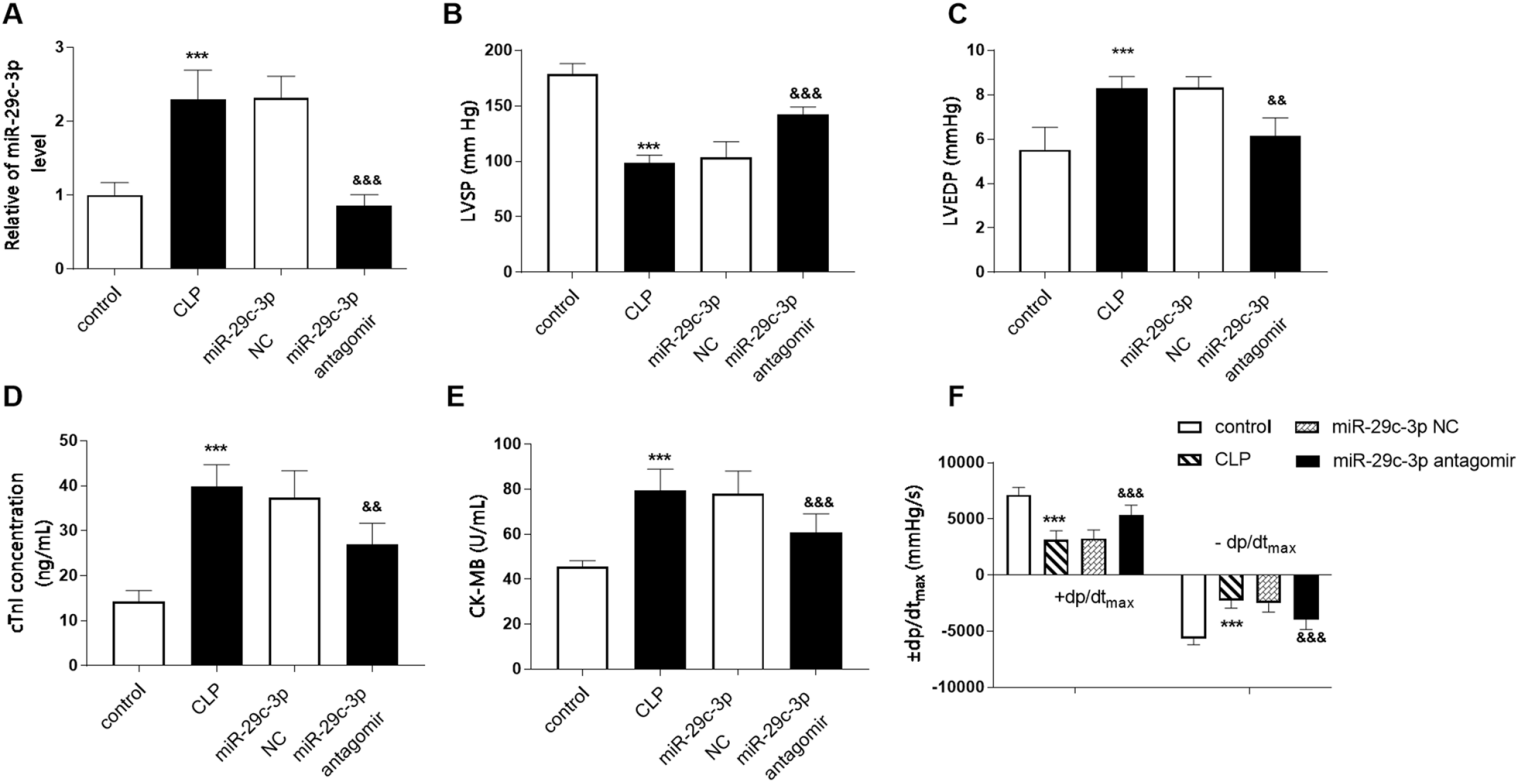
Influences of miR-29c-3p on cardiac function in sepsis rat models. **A** Serum level of miR-29c-3p in different model groups. The changes of **B** LVSP, **C** LVEDP, **D** cTnI, **E** CK-MB and **F** ± d*p*/d*t*_max_ in different model groups. (***P < 0.001 vs. control group, ^$$$^*P* < 0.001, ^$$^*P* < 0.01 vs. CLP group)

The effect of miR-29c-3p on cardiac function in patients with sepsis was studied by constructing an animal model of sepsis in vitro. miR-29c-3p level in serum of CLP model rats was significantly increased (Fig. 3A, *P* < 0.001), which showed a consistent trend with the results shown in Fig. 1. Besides, miR-29c-3p level in CLP rats was inhibited after intravenous administration of miR-29c-3p antagomir. Compared with the control group, LVSP and + d*p*/d*t*_max_ in the CLP group diminished, while LVEDP and − d*p*/d*t*_max_ enhanced significantly, accompanied by the increase of serum cTnI and CK-MB in the rats after monitoring the cardiac function, which indicated that cardiac dysfunction occurred in the sepsis rat model (Fig. 3B–F, *P* < 0.001). Moreover, we also noted that inhibition of miR-29c-3p after the injection of miR-29c-3p antagomir significantly improved cardiac function in CLP rats, which was mainly presented by increased LVSP and + d*p*/d*t*_max_, and decreased LVEDP and − d*p*/d*t*_max_, as well as the level of cTnI and CK-MB. In view of these results, we believe that miR-29c-3p is involved in the regulation of septic cardiac dysfunction.

### Influences of miR-29c-3p on inflammation in sepsis rats

**Fig. 4:**
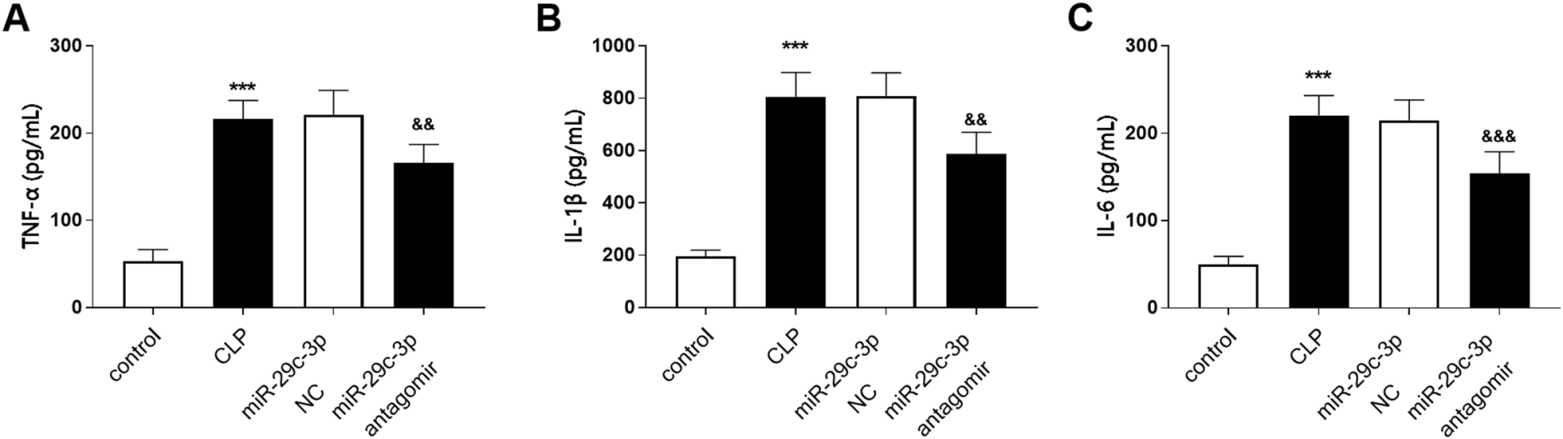
Effects of miR-29c-3p on inflammatory cytokines in sepsis rats. Serum level of **A** TNF-α, **B** IL-1β, and **C** IL-6 in sepsis rat models. (***P < 0.001 vs. control group, ^$$$^*P* < 0.001, ^$$^*P* < 0.01 vs. CLP group)

The levels of inflammatory indicators in serum were determined in sepsis rat models. As shown in Fig. 4, the levels of TNF-α, IL-1β and IL-6 were enhanced in the CLP group compared with the control group, while the downregulation of miR-29c-3p level could effectively reduce the levels of these inflammatory factors (Fig. 4A–C, *P* < 0.01). These results suggested that inhibition of miR-29c-3p expression could down-regulate the level of sepsis induced inflammatory response.

## Discussion

A rapidly developing systemic inflammatory response syndrome caused by infection is known as sepsis [14]. In clinical practice, sepsis can eventually develop into septic shock or multiple organ dysfunction syndrome if effective measures are not taken to control the early and continuous development of sepsis [15]. For the current diagnosis of sepsis, commonly used clinical biomarkers involve CRP, WBC, erythrocyte sedimentation rate (ESR), and procalcitonin (PCT) [16, 17]. However, it is found that the above biomarkers are far from playing the expected role in practical application. Therefore, it is still necessary to continue to search for new and specific biomarkers to serve the clinical practice.

The ideal biomarker should have high sensitivity and specificity and should also be easy to obtain and quickly detect. miRNAs are widely present in body fluids, including blood and urine, and their highly stable characteristics in circulation make them capable of being used as biomarkers for clinical reference. As a tumor suppressor in the miR-29 family, miR-28c-3p, located at chromosome 1q32.3 [18], has been proved to be down-regulated in many solid tumors, such as gastric cancer, colorectal cancer, gallbladder cancer, etc. [19–21]. Fang et al.’s study showed that restraint of miR-29c-3p expression in laryngeal squamous cell carcinoma was significantly correlated with lymphatic metastasis and cancer typing [22]. In addition, Wu et al. reported that miR-29c-3p level was attenuated in patients with Alzheimer's disease. In our study, it was found that miR-29c-3p level was up-regulated in sepsis patients, and it was closely related to the clinicopathological indicators of sepsis patients. Meanwhile, the ROC curve proved that the level of miR-29c-3p had high sensitivity and specificity in the diagnosis of sepsis. Furthermore, after classifying sepsis patients according to whether cardiac dysfunction has occurred, we found that serum level of miR-29c-3p in sepsis patients with cardiac dysfunction was generally higher than that in sepsis patients without cardiac dysfunction. These findings suggested that high miR-29c-3p levels were correlated with the severity of sepsis.

According to statistics, sepsis patients with cardiac dysfunction have an increased mortality rate of 70% to 90% compared with patients who had no cardiac dysfunction [23]. The status of cardiovascular system and cardiac function in patients has always been the focus of basic clinical research during sepsis [24]. Studies have shown that hypoxia of the myocardium is a possible cause or sequelae of heart failure, it often occurs when coronary perfusion is reduced under high cardiac congestion pressure, including sepsis and high-altitude conditions [25]. Guo et al.’s study exhibited that the level of miR-29c-3p in the right ventricle of hypoxic mice was significantly up-regulated over time [26]. Huang et al. showed that the level of miR-29c-3p was positively correlated with the degree of left ventricular hypertrophy in patients with systemic arterial hypertension [27]. Ye et al. revealed that miR-29c-3p promoted myocardial apoptosis by targeting MCL1 [28]. In this study, Logistics regression analysis proved that miR-29c-3p was an independent factor for cardiac dysfunction in sepsis patients. We evaluated the role of miR-29c-3p in sepsis-induced cardiac dysfunction by building a rat sepsis model. Our studies have shown that the serum miR-29c-3p expression of sepsis rat model was increased, and severe cardiac dysfunction appeared. However, it significantly improved the cardiac function of sepsis rats by suppressing miR-29c-3p expression. Our results are consistent with those of previous published studies. Therefore, we speculated that miR-29c-3p is involved in the regulation of cardiac function in sepsis rats. The relationship between miR-29 family and inflammatory response has been confirmed in many studies. For example, Chen et al. reported that the restoration of miR-29 levels in diabetic nephropathy inhibited the TGF-β/Smad3 pathway, thereby reducing the accumulation of collagen matrix and inflammatory response [29]. Another study by Sun et al. has shown that miR-29c promotes inflammation in Type 2 diabetes mellitus by recruiting and activating circulating monocytes and macrophages [30]. Our study found that serum inflammatory cytokines were significantly enhanced in the sepsis rat model, while inhibition of miR-29c-3p expression decreased the level of inflammatory cytokines.

Based on the analysis of the above results, we think that the anti-inflammatory effect demonstrated by antagonistic miR-29c-3p may be related to its protective effect on cardiac function. However, the mechanism of miR-29c-3p regulating cardiac function and inflammatory response is still unknown. These tissues as the limitations of this study still need further exploration.

In general, serum miR-29c-3p level was increased in sepsis patients. And miR-29c-3p expression was up-regulated in sepsis patients with cardiac dysfunction than in sepsis patients without cardiac dysfunction. miR-29c-3p level has high clinical value for the diagnosis of sepsis. Animal model experiments have confirmed that inhibition of miR-29c-3p improved sepsis-induced cardiac dysfunction and inflammatory response.

## Data Availability

The datasets used and/or analysed during the current study are available from the corresponding author on reasonable request.

## Abbreviations

AOSD: Adult-onset Still’ s disease
APACHE-II: Acute physiological and chronic health
BMI: Body mass index
CK-MB: Creative kinase isoenzyme MB
CLP: Cecal ligation and puncture
CRP: C-reactive protein
cTnI: Cardiac troponin I
ESR: Erythrocyte sedimentation rate
ICU: Intensive care unit
IL-6: Interleukin 6
IL-1β: Interleukin 1β
LVSP: Left ventricular systolic pressure
LVEDP: Left ventricular end diastolic pressure
PCT: Procalcitonin
SOFA: Score and organ dysfunction
TNF-α: Tumor necrosis factor α
WBC: White blood cell
3′-UTR: 3′-Non-transcriptional regions
